# Prognostic Impact of Immune-Related Adverse Events on Combination Immune Checkpoint/Tyrosine Kinase Inhibition for Metastatic Renal Cancer

**DOI:** 10.1158/2767-9764.CRC-25-0337

**Published:** 2025-09-19

**Authors:** Bunpei Isoda, Masanobu Shiga, Shuya Kandori, Shota Takahashi, Akifumi Omiya, Tomoki Ishida, Kotoe Matsuda, Hiromichi Sakurai, Bryan J. Mathis, Ken Tanaka, Manabu Komine, Masahiro Iinuma, Akira Joraku, Hiromitsu Negoro, Masakazu Tsutsumi, Takamitsu Inoue, Jun Miyazaki, Hiroyuki Nishiyama

**Affiliations:** 1 Department of Urology, Institute of Medicine, University of Tsukuba, Tsukuba, Japan.; 2 Department of Urology, Ibaraki Prefectural Central Hospital, Kasama, Japan.; 3 Department of Urology, Hitachi General Hospital, Hitachi, Japan.; 4 Department of Cardiovascular Surgery, Institute of Medicine, University of Tsukuba, Tsukuba, Japan.; 5 Department of Urology, Tsukuba Medical Center Hospital, Tsukuba, Japan.; 6 Department of Urology, National Hospital Organization Mito Medical Center, Mito, Japan.; 7 Department of Urology, International University of Health and Welfare Narita Hospital, Narita, Japan.

## Abstract

Immune checkpoint inhibitor (ICI) combinations and tyrosine kinase inhibitor (TKI) use are standard for metastatic renal cell carcinoma (mRCC), leading to improved outcomes. However, due to a lack of predictive biomarkers, the presence or absence of immune-related adverse events (irAE) is currently used as a predictive factor in clinical practice. To elucidate the impact of irAEs on efficacy, a cohort of patients with mRCC who received ICI-based combination therapy as initial treatment was analyzed. Patients were divided into two groups: those who received dual-ICI therapy (ICI–ICI, *N* = 55) or ICI and TKI therapy (ICI–TKI, *N* = 55). Subsequent to this initial categorization, each group was further subdivided based on the presence or absence of irAEs. In the ICI–ICI group, patients with irAEs exhibited significantly prolonged overall survival (OS) and progression-free survival [PFS; OS (median): not reached vs. 17.9 months, *P* = 0.03/PFS (median): 51.4 vs. 5.8 months, *P* < 0.01]. Conversely, no such correlation was observed between irAEs and OS/PFS in the ICI–TKI group. [OS (median): 26.3 months vs. not reached, *P* = 0.73/PFS (median): 16.8 months vs. 11.9 months, *P* = 0.38] Furthermore, treatment discontinuation due to AEs accounted for 65% (*N* = 32) in the ICI–ICI group and 57% (*N* = 24) in the ICI–TKI group, with a slightly higher tendency observed in the ICI–ICI group. These findings suggest that the prognostic impact of irAEs may differ depending on the treatment combination, and further basic research is needed to elucidate the underlying mechanisms.

**Significance:**

In patients with mRCC who received ICI–ICI, the occurrence of irAEs was associated with improved survival outcomes, whereas no such association was observed in patients who received ICI–TKI. This suggests that irAEs may reflect the favorable immune response specific to ICI–ICI therapy, whereas differences in the tumor microenvironment, particularly involving neutrophils, in ICI–TKI patients may influence treatment response.

## Introduction

Metastatic renal cell carcinoma (mRCC) treatment has been significantly advanced with the advent of immune checkpoint inhibitors (ICI) that remove blocks on cytotoxic CD8^+^ T-cell antitumor activity. Dual-ICI (ICI–ICI) or ICI plus tyrosine kinase inhibitor (ICI–TKI) combination therapies have become the first-line treatment for mRCC, and clinical trials have indicated a median overall survival (mOS) of around 4 years (1–5), superior to TKI or ICI monotherapy.

However, neither reports directly comparing these regimens nor clear criteria for their use are currently published. In addition, there are no currently reported biomarkers that can reliably predict ICI response. Therefore, it is empirically accepted in clinical practice to correlate immune-related adverse events (irAE) with treatment efficacy, and occurrence rates are correlated with positive outcomes in other cancers, including RCC (6–8). Even in reports limited to patients with mRCC treated with nivolumab/ipilimumab, irAE occurrence is considered a proxy for favorable prognoses (9). However, the impact of irAEs on treatment efficacy may differ for ICI–TKIs, which combine different mechanisms of action. Because TKIs inhibit tumor growth, as opposed to enhancing the action of CD8^+^ T cells, newly discovered biomarkers for TKI effect (such as EGFR and hypomethylation) in the presence of ICIs remain unverified in mRCC (10).

In this study, we retrospectively analyzed patients with mRCC who received ICI–ICI or ICI–TKI as first-line treatment and evaluated the relationship between irAEs and treatment efficacy. By comparing survival outcomes in each treatment group with irAE occurrence, we aimed to clarify the role of irAEs as a prognostic factor in the treatment of mRCC.

## Materials and Methods

### Patients

From December 2015 to February 2025, 165 patients with mRCC started treatment with ICI–ICI or ICI–TKI as first-line therapy at six Japanese institutions. In accordance with previous representative clinical trials, the study was limited to patients with clear cell carcinoma. Additionally, cases were excluded if important data such as survival outcomes were missing or if the initial imaging evaluation could not be performed due to the short duration of treatment from the start of therapy. Furthermore, because ICI–ICI is not indicated for patients classified as “favorable” in the International Metastatic RCC Database Consortium (IMDC) classification, cases classified as “favorable” in the IMDC classification were excluded to ensure background consistency, resulting in a final analysis of 110 cases. We collected patient background information and treatment outcomes for these patients. The included patients were divided into two groups: ICI–ICI or ICI–TKI. Furthermore, each group was subdivided into two groups according to the presence or absence of irAEs (irAE+ or irAE−). For the purposes of this study, irAEs were defined as AEs that may involve immunologic mechanisms and may require intervention with immunosuppressive therapy (as previously reported; refs. 11–13). The severity of AEs was assessed based on the Common Terminology Criteria for Adverse Events v5.0. The determination of AEs was made primarily by the attending urologist, who consulted with other specialists as necessary. The choice of treatment regimen was left to the discretion of the attending urologist. All treatments were administered in accordance with the approved dosage and package insert in Japan.

### Definition and evaluation method of survival indices

OS was defined as the time from the start of treatment to the confirmation of death. For patients who remained alive, data were censored at the last confirmed survival timepoint. Progression-free survival (PFS) was defined as the time from the start of treatment to the confirmation of disease progression or death. For patients who did not experience disease progression or death, data were censored at the last confirmed PFS timepoint. Tumor assessments were performed according to the RECIST v1.1 (14) for determining the efficacy of treatment for solid tumors. Based on the clinical judgment of each attending urologist, imaging tests such as CT and MRI were performed at appropriate times. The data cutoff date was February 28, 2025.

### Statistical analyses

All statistical analyses were performed using R4.2.3 (R Development Core Team, RRID: SCR_001905). The significance of any differences between groups was assessed by the Wilcoxon rank-sum test, Pearson *χ*^2^ test, or Fisher exact test. *P* values < 0.05 were considered statistically significant. Survival curves for OS and PFS were estimated using the Kaplan–Meier method, and comparisons between groups were performed with the log-rank test. Median survival times were reported, and HRs with corresponding 95% confidence intervals (CI) were calculated. Multivariate analyses were conducted using Cox proportional hazards regression models to adjust for potential confounding factors.

### Ethics approval

The studies involving human participants were reviewed and approved by Tsukuba University Hospital Institutional Review Board (#R05-057). Written informed consent was obtained from all patients prior to enrollment. All procedures were conducted in accordance with the Declaration of Helsinki and other relevant ethical guidelines.

### Data availability

The data in this study are available upon request from the corresponding author.

## Result

### Patient characteristics

Of the 110 cases, 55 were in the ICI–ICI group and 55 were in the ICI–TKI group. Whereas there were more elderly patients in the ICI–TKI group (Table 1), there were no significant differences between the two groups in terms of IMDC classification or metastatic site. Within the ICI–TKI group, cabozantinib/nivolumab combination therapy was most commonly used (49%), followed by lenvatinib/pembrolizumab combination therapy (25%), axitinib/pembrolizumab combination therapy (15%), and axitinib/avelumab combination therapy (11%).

**Table 1.** Patient characteristics.

Characteristic	Treatment type			*P* value^b^
	All *N* = 110^a^	ICI–ICI *N* = 55 (50%)^a^	ICI–TKI *N* = 55 (50%)^a^	
Age	70 (65–75)	68 (60–75)	72 (67–77)	0.019
Sex	​	​	​	0.61
Male	92 (84%)	45 (82%)	47 (85%)	​
Female	18 (16%)	10 (18%)	8 (15%)	​
Metastasis timing	​	​	​	0.081
Metachronous	45 (41%)	27 (49%)	18 (33%)	​
Synchronous	65 (59%)	28 (51%)	37 (67%)	​
Regimen	​	​	​	<0.0001
Ipi + Nivo	55 (50%)	55 (100%)	0 (0%)	​
Cabo + Nivo	27 (25%)	0 (0%)	27 (49%)	​
Len + Pem	14 (13%)	0 (0%)	14 (25%)	​
Axi + Pem	8 (7.3%)	0 (0%)	8 (15%)	​
Axi + Ave	6 (5.5%)	0 (0%)	6 (11%)	​
IMDC	​	​	​	0.66
Intermediate	82 (75%)	42 (76%)	40 (73%)	​
Poor	28 (25%)	13 (24%)	15 (27%)	​
Lung metastasis	​	​	​	0.84
Absent	35 (32%)	17 (31%)	18 (33%)	​
Present	75 (68%)	38 (69%)	37 (67%)	​
Lymph node metastasis	​	​	​	0.84
Absent	77 (70%)	39 (71%)	38 (69%)	​
Present	33 (30%)	16 (29%)	17 (31%)	​
Liver metastasis	​	​	​	>0.99
Absent	102 (93%)	51 (93%)	51 (93%)	​
Present	8 (7.3%)	4 (7.3%)	4 (7.3%)	​
Bone metastasis	​	​	​	0.37
Absent	84 (76%)	40 (73%)	44 (80%)	​
Present	26 (24%)	15 (27%)	11 (20%)	​
Brain metastasis	​	​	​	>0.99
Absent	107 (97%)	54 (98%)	53 (96%)	​
Present	3 (2.7%)	1 (1.8%)	2 (3.6%)	​

a Median (IQR); *n* (%).

b Wilcoxon rank-sum test; Pearson *χ*^2^ test; Fisher exact test.

Abbreviations: Ave, avelumab; Axi, axitinib; Cabo, cabozantinib; Ipi, ipilimumab; Len, lenvatinib; Nivo, nivolumab; Pem, pembrolizumab.

### Responses and survival outcomes

**Figure 1.**
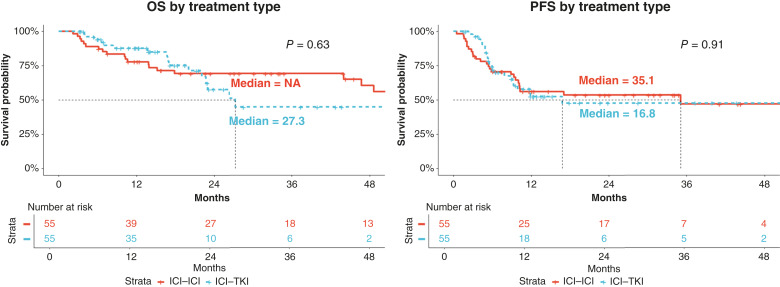
Kaplan–Meier curves by treatment. The red line represents the ICI–ICI group, and the blue line represents the ICI–TKI group.

The median observation period was 28.9 months (range, 3.2–110.1 months), and there were no significant differences in OS or PFS by treatment (Fig. 1). The mOS was not reached in the ICI–ICI group and 27.3 months in the ICI–TKI group (*P* = 0.63). The median PFS (mPFS) was 35.1 months in the ICI–ICI group and 16.8 months in the ICI–TKI group (*P* = 0.91). The response rates for ICI–ICI therapy were as follows: complete response (CR) 8.8% (*N* = 5), partial response (PR) 35.1% (*N* = 20), stable disease (SD) 33.3% (*N* = 19), and progressive disease (PD) 22.8% (*N* = 13). For ICI–TKI therapy, the response rates were as follows: CR 12.3% (*N* = 7), PR 31.5% (*N* = 18), SD 50.9% (*N* = 29), and PD 5.3% (*N* = 3). The overall response rate (CR and PR) was 45% (*N* = 25) in the ICI–ICI group and 44% (*N* = 24) in the ICI–TKI group; however, the ICI–TKI group had more SD and PD patients than the ICI–ICI group.

Regarding treatment continuation, 11% (*N* = 6) of the ICI–ICI group continued treatment whereas 89% (*N* = 49) discontinued treatment for various reasons. In the ICI–TKI group, 24% (*N* = 13) continued treatment whereas 24% (*N* = 76) discontinued treatment. In the ICI–ICI group, 65% (*N* = 32) discontinued treatment due to AEs and 29% (*N* = 14) discontinued treatment due to PD. In contrast, in the ICI–TKI group, 57% (*N* = 24) discontinued treatment due to AEs and 33% (*N* = 14) discontinued treatment due to PD (Supplementary Table S1).

### irAEs

The incidence of irAEs in all grades was significantly higher in the ICI–ICI group [*N* = 42 (76%)] than in the ICI–-TKI group [*N* = 31 (56%); *P* = 0.026], but there was no significant difference in treatment-related irAEs of grade 3 or higher (Table 2).

**Table 2.** Status of irAE.

Characteristic	Treatment type			*P* value^b^
	All *N* = 110^a^	ICI–ICI *N* = 55 (50%)^a^	ICI–TKI *N* = 55 (50%)^a^	
All irAEs	​	​	​	​
All grade	73 (66%)	42 (76%)	31 (56%)	0.026
More than G3	33 (30%)	16 (29%)	17 (31%)	0.84
Thyroid	​	​	​	​
All grade	28 (25%)	17 (31%)	11 (20%)	0.19
More than G3	6 (5.5%)	4 (7.3%)	2 (3.6%)	0.68
Adrenal	​	​	​	​
All grade	13 (12%)	10 (18%)	3 (5.5%)	0.039
More than G3	6 (5.5%)	4 (7.3%)	2 (3.6%)	0.68
Pituitary	​	​	​	​
All grade	8 (7.3%)	8 (15%)	0 (0%)	0.0059
More than G3	4 (3.6%)	4 (7.3%)	0 (0%)	0.12
Hepatitis	​	​	​	​
All grade	13 (12%)	5 (9.1%)	8 (15%)	0.38
More than G3	11 (10%)	3 (5.5%)	8 (15%)	0.11
Rush	​	​	​	​
All grade	18 (16%)	9 (16%)	9 (16%)	>0.99
More than G3	5 (4.5%)	2 (3.6%)	3 (5.5%)	>0.99

a *n* (%).

b Pearson *χ*^2^ test; Fisher exact test.

Abbreviation: G3, grade 3.

With regard to individual irAEs, pituitary insufficiency of all grades occurred more frequently in the ICI–ICI group, regardless of grade [ICI–ICI group: *N* = 8 (15%)/ICI–TKI group: *N* = 0 (0%), *P* = 0.0059], and adrenal insufficiency of all grades occurred more frequently in the ICI–ICI group [ICI–ICI group: *N* = 10 (18%)/ICI–TKI group: *N* = 3 (5.5%), *P* = 0.039]. Other irAEs occurred equally between the two groups.

### Survival analysis by the presence or absence of irAEs

**Figure 2.**
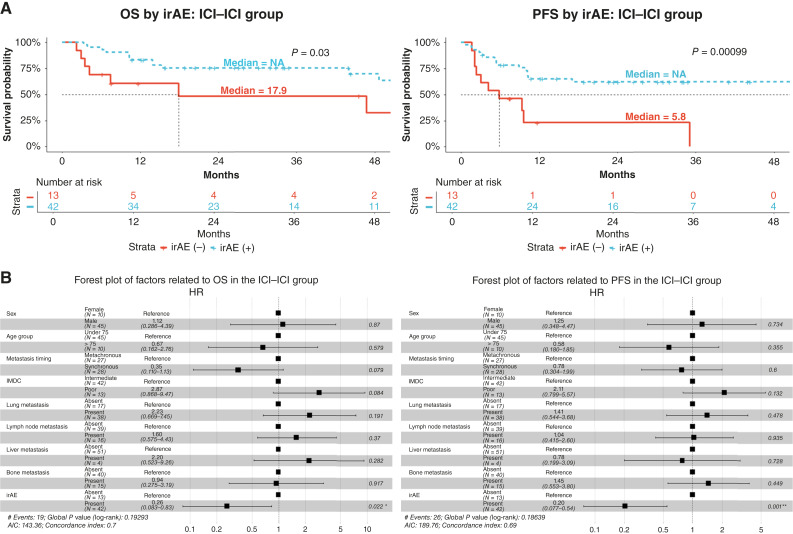
Survival outcomes and multivariable analysis of prognostic factors in the ICI–ICI group. **A,** Kaplan–Meier curves for OS and PFS. The results of a survival analysis conducted on the ICI–ICI group with and without irAEs. The red line represents the patient group without irAEs, and the blue line represents the patient group with irAEs. **B,** Multivariable analysis of OS and PFS of the ICI–ICI group. NA, not available.

In the ICI–ICI group, no significant differences were observed in patient characteristics between the two groups divided according to the presence or absence of irAEs (Table 3). However, in the OS/PFS analysis for the ICI–ICI group, both OS and PFS were significantly longer in the irAE+ group (Fig. 2A). The mOS was not reached in the irAE+ group and was 17.9 months in the irAE− group, but the mPFS was 51.4 months in the irAE+ group and 5.8 months in the irAE− group. We also performed multivariable analysis of each variable using the Cox proportional hazards model to assess the impact of these factors on survival (Fig. 2B). In the ICI–ICI group, regarding OS, there was a significant difference only in irAEs [HR 0.26 (95% CI, 0.083–0.83), *P* = 0.022] but no difference in other variables. Regarding PFS, there was a significant difference only in irAEs [HR 0.20 (95% CI, 0.077–0.54), *P* = 0.001], with no difference in other variables.

**Table 3.** Patient characteristics of the ICI–ICI group.

Characteristic	ICI–ICI group Presence or absence of irAE			*P* value^b^
	All *N* = 55^a^	Absent *N* = 13 (50%)^a^	Present *N* = 42 (76%)^a^	
Age	68 (60–75)	68 (66–78)	67 (59–74)	0.25
Sex	​	​	​	0.42
Male	45 (82%)	12 (92%)	33 (79%)	​
Female	10 (18%)	1 (7.7%)	9 (21%)	​
Metastasis timing	​	​	​	0.69
Metachronous	27 (49%)	7 (54%)	20 (48%)	​
Synchronous	28 (51%)	6 (46%)	22 (52%)	​
IMDC	​	​	​	0.71
Intermediate	42 (76%)	11 (85%)	31 (74%)	​
Poor	13 (24%)	2 (15%)	11 (26%)	​
Lung metastasis	​	​	​	0.19
Absent	17 (31%)	6 (46%)	11 (26%)	​
Present	38 (69%)	7 (54%)	31 (74%)	​
Lymph node metastasis	​	​	​	0.73
Absent	39 (71%)	10 (77%)	29 (69%)	​
Present	16 (29%)	3 (23%)	13 (31%)	​
Liver metastasis	​	​	​	0.23
Absent	51 (93%)	11 (85%)	40 (95%)	​
Present	4 (7.3%)	2 (15%)	2 (4.8%)	​
Bone metastasis	​	​	​	0.31
Absent	40 (73%)	8 (62%)	32 (76%)	​
Present	15 (27%)	5 (38%)	10 (24%)	​
Brain metastasis	​	​	​	>0.99
Absent	54 (98%)	13 (100%)	41 (98%)	​
Present	1 (1.8%)	0 (0%)	1 (2.4%)	​

a Median (IQR); *n* (%).

b Wilcoxon rank-sum test; Pearson *χ*^2^ test; Fisher exact test.

**Figure 3.**
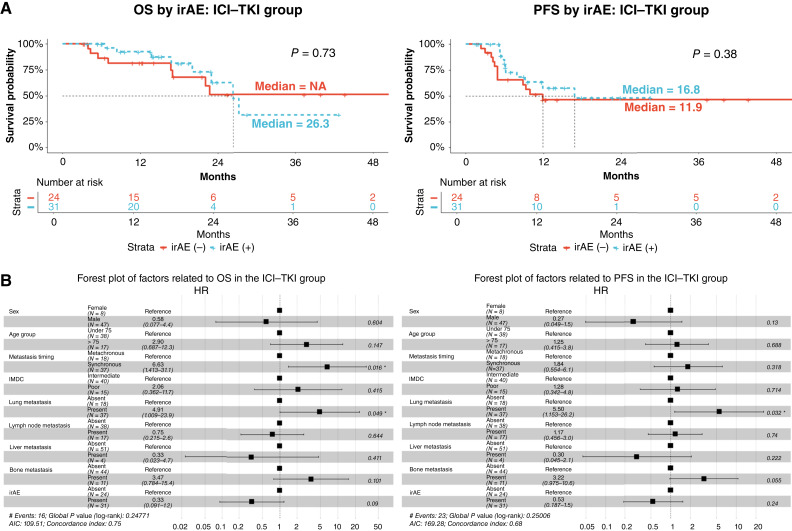
Survival outcomes and multivariable analysis of prognostic factors in the ICI–TKI group. **A,** Kaplan–Meier curves for OS and PFS. The results of a survival analysis conducted on the ICI–TKI group with and without irAEs. The red line represents the patient group without irAEs, and the blue line represents the patient group with irAEs. **B,** Multivariable analysis of OS and PFS of the ICI–TKI group. NA, not available.

Within the ICI–TKI group, the irAE+ group was predominantly older, but there was no clear difference in any other variable (Table 4). In the analysis of OS/PFS in the ICI–TKI group, there was no clear difference in the presence or absence of irAEs (Fig. 3A). The mOS was 26.3 months in the irAE+ group and was not reached in the irAE− group. The mPFS was 16.8 months in the irAE+ group and 11.9 months in the irAE− group. The results of the multivariable analysis of each factor using the Cox proportional hazards model showed that, in the ICI–TKI group with regard to OS, there were significant differences in metastasis timing [HR 6.63 (95% CI, 1.413–31.1), *P* = 0.016] and lung metastasis [HR 4.91 (95% Cl, 1.009–23.9), *P* = 0.049]. Regarding PFS, there was a significant difference only in lung metastasis [HR 5.50 (95% CI, 1.153–26.2), *P* = 0.032] and no differences in other variables (Fig. 3B).

**Table 4.** Patient characteristics of the ICI–TKI group.

Characteristic	ICI–TKI group Presence or absence of irAE			*P* value^b^
	All *N* = 55^a^	Absent *N* = 24 (44%)^a^	Present *N* = 31 (56%)^a^	
Age	72 (67–77)	68 (62–76)	72 (70–77)	0.083
Sex	​	​	​	0.28
Male	47 (85%)	19 (79%)	28 (90%)	​
Female	8 (15%)	5 (21%)	3 (9.7%)	​
Metastasis timing	​	​	​	0.21
Metachronous	18 (33%)	10 (42%)	8 (26%)	​
Synchronous	37 (67%)	14 (58%)	23 (74%)	​
IMDC	​	​	​	0.13
Intermediate	40 (73%)	15 (63%)	25 (81%)	​
Poor	15 (27%)	9 (38%)	6 (19%)	​
Lung metastasis	​	​	​	0.93
Absent	18 (33%)	8 (33%)	10 (32%)	​
Present	37 (67%)	16 (67%)	21 (68%)	​
Lymph node metastasis	​	​	​	0.73
Absent	38 (69%)	16 (67%)	22 (71%)	​
Present	17 (31%)	8 (33%)	9 (29%)	​
Liver metastasis	​	​	​	>0.99
Absent	51 (93%)	22 (92%)	29 (94%)	​
Present	4 (7.3%)	2 (8.3%)	2 (6.5%)	​
Bone metastasis	​	​	​	0.74
Absent	44 (80%)	20 (83%)	24 (77%)	​
Present	11 (20%)	4 (17%)	7 (23%)	​
Brain metastasis	​	​	​	>0.99
Absent	53 (96%)	23 (96%)	30 (97%)	​
Present	2 (3.6%)	1 (4.2%)	1 (3.2%)	​

a Median (IQR); *n* (%).

b Wilcoxon rank-sum test; Pearson *χ*^2^ test; Fisher exact test.

## Discussion

The advent of immunotherapy has improved the prognosis of mRCC, with ICI becoming a cornerstone of most treatment strategies. According to the National Comprehensive Cancer Network guidelines (https://www.nccn.org/guidelines/guidelines-detail?category=1&id=1440, RRID: SCR_012959), ICI–ICI or ICI–TKI are first-line treatment options.

The duration of response for ICI–TKI is about 2 years (4, 15), but ICI–ICI may achieve a longer duration of response in patients with mRCC (16). On the other hand, dual-ICI is associated with PD in approximately 20% of patients in the early stages of treatment (1), and although direct comparisons have not been made, ICI–TKI tends to have a higher proportion of PD than dual-ICI (1, 4). In our cohort, similar to previous reports, the proportion of PD was higher in the ICI–ICI group compared with the ICI–TKI group. However, the mOS and mPFS were similar to previous reports. This suggests that optimal treatment selection and timing probably matter most in achieving favorable outcomes. Choosing optimal treatment combinations, however, remains difficult due to a lack of reliable biomarkers.

Whereas there are diverse reports of numerous biomarkers for treatment response (e.g., microbiome, transcriptome, EGFR, etc.)(17), all remain clinically unestablished. For this reason, the presence or absence of irAEs as a proxy of immune status and subsequent antitumor effect is used to empirically predict the efficacy of ICI treatment (6, 7). Such reports have been made for multiple cancer types, and the pattern is thought to also hold for renal cancer (8, 9). However, in our irAE ± cohorts, different results were obtained. In the ICI–ICI group, the presence or absence of irAEs had an impact on survival, whereas in the ICI–TKI group, there was no association observed.

Our contrasting results may be due to unclear mechanisms that relate to simultaneous activation of complex immunogenic or immunoregulatory pathways. One such theory, the antigen mimicry theory, posits that, in a cancer such as melanoma, multiple tumor epitopes common to normal melanocytes dilute antitumor activity (18, 19). In addition, it has been reported in melanoma and non–small cell lung cancer that there is a significant overlap between irAE lesions and T cells that have infiltrated the tumor (20). These findings suggest that the release of shared antigens by ICIs may trigger secondary immune responses to the host’s antigenic response sites. Furthermore, it has also been suggested that abnormal regulation of humoral immunity may also be involved as the PD-1 signaling pathway affected by ICIs regulates B-cell activation in both T cell–dependent and –independent ways (21, 22). Such mechanisms may explain the observed positive correlation between irAEs and the therapeutic efficacy of ICIs.

The available evidence does not provide a clear explanation of the discrepancy observed in our ICI–TKI group. However, TKI targets VEGFR, a crucial factor in RCC that promotes endothelial cell migration, proliferation, and survival, by blocking autophosphorylation, thereby inhibiting these processes (23–25). All TKIs used in mRCC exert their direct antitumor effects by acting on VEGFR but also decrease regulatory T-cell populations and may have some effect on naïve/effector T-cell population balance in addition to skewing macrophage population (26, 27). Given the nature of the treatment regimen, which consists of two drugs with distinct mechanisms of action (ICIs are immune-specific whereas TKIs are systemic in effect), it is therefore challenging to ascertain which agent predominantly contributes to the therapeutic effect. Even if irAEs have been identified as a potential predictive marker for the efficacy of ICIs, they may not necessarily reflect the efficacy of TKIs, and this could explain the observed absence of correlation between the incidence of irAEs and the overall treatment response. In addition, a report on the combination therapy of cabozantinib and anti–PD-1 antibody for hepatocellular carcinoma found that the suppression of CD8^+^ T-cell infiltration into tumors was ablated but there was an increase in neutrophil infiltration (28). That report suggests a bridging role for neutrophils between innate and acquired immunity that may synergistically intensify antitumor responses. If this phenomenon is also reproduced in mRCC, it is possible that the antitumor effect of ICI–TKI combination therapy is amplified through neutrophils rather than T cells or macrophages (29). This is significant because T cells are believed to play a role in the development of irAEs. As such, the presence or absence of irAEs may not be a reliable indicator of the efficacy of ICI–TKI treatment.

Considering these findings, it can be posited that the occurrence of irAE, via changes in T-cell effector populations, may be associated with treatment response in dual-ICI therapy. Therefore, it may be beneficial to continue treatment while managing adverse effects to the greatest extent possible. On the other hand, as there may be no correlation between the occurrence of irAEs and the efficacy of potentially neutrophil-centric ICI–TKI therapy, it may be essential to consider changing the treatment regimen more aggressively in cases in which irAE management is difficult.

There are several limitations to this study. First, the retrospective nature may have introduced an unavoidable bias in patient selection, treatment selection, and/or findings obtained from the analysis. In particular, treatment selection was left to the discretion of each attending urologist, and there may be potential selection bias. Second, not all irAEs (especially very mild or transient irAEs) were recognized, and there is a possibility that unrecorded irAEs affected the analysis. In addition, whether AEs were caused by ICIs or TKIs was determined comprehensively by the attending urologist based on the course of treatment and test results, which may have introduced bias. Third, the interval between imaging tests was irregular, and this may have affected the survival analysis. It should also be noted that ICI–TKI was not covered by health insurance in Japan until 2019, well after the approval of dual-ICI in 2018.

Our analysis suggests that the presence or absence of irAEs may be used to predict treatment efficacy in dual-ICI therapy for mRCC. On the other hand, no relationship was found between treatment efficacy and irAEs in ICI–TKI. In the future, fundamental mechanisms linking irAEs and treatment efficacy will require surveillance of macrophages, neutrophils, and T cells to create patterns that may be used as potential biomarkers for response. If such research progresses, it is hoped that more efficient biomarkers and treatment efficacy prediction models will be developed.

## Supplementary Material

Supplemental Table 1 Supplementary Table 1
